# Bovine milk microbiome: a more complex issue than expected

**DOI:** 10.1186/s13567-019-0662-y

**Published:** 2019-06-06

**Authors:** Suvi Taponen, David McGuinness, Heidi Hiitiö, Heli Simojoki, Ruth Zadoks, Satu Pyörälä

**Affiliations:** 1 Department of Production Animal Medicine, Faculty of Veterinary Medicine, University of Helsinki, Helsinki, Finland; 2 Glasgow Polyomics, College of Medical, Veterinary and Life Sciences, University of Glasgow, Glasgow, UK; 3 Institute of Biodiversity, Animal Health and Comparative Medicine, College of Medical, Veterinary and Life Sciences, University of Glasgow, Glasgow, UK

## Abstract

The aim of this study was to analyze bacterial profiles of bovine mastitic milk samples and samples from healthy quarters using Next Generation Sequencing of amplicons from 16S rRNA genes and to compare results with microbiological results by PCR assays of the same samples. A total of 49 samples were collected from one single dairy herd during the same day. The samples were divided in two sample sets, which were used in this study. The DNA extraction as well as the library preparation and sequencing of these two sets were performed separately, and results of the two datasets were then compared. The vast majority of genera detected appeared with low read numbers and/or in only a few samples. Results of PCR and microbiome analyses of samples infected with major pathogens *Staphylococcus aureus* or *Streptococcus uberis* were consistent as these genera also covered the majority of reads detected in the microbiome analysis. Analysis of alpha diversity revealed a much higher species richness in set 1 than in set 2. The dominating bacterial genera with the highest read numbers clearly differed between datasets, especially in PCR negative samples and samples positive for minor pathogens. In addition to this, linear discriminant analysis (LDA) was conducted between the two sets to identify significantly different genera/family level microbes. The genus *Methylobacterium* was much more common in set 2 compared to set 1, and genus *Streptococcus* more common in set 1. Our results indicate amplification of contaminating bacteria in excess in samples with no or minor amounts of pathogen DNA in dataset 2. There is a need for critical assessment of results of milk microbiome analyses.

## Introduction

Inflammation of the mammary gland, mastitis, is most commonly caused by intramammary infection (IMI) derived from bacteria. Approximately 140 bacterial species have been isolated by conventional culture in milk samples taken from infected bovine mammary quarters [1]. The most common pathogen genera isolated in mastitic milk samples are staphylococci, enterobacteria and streptococci, which cause the great majority of IMIs [2–4]. Udder pathogens have traditionally been divided into major and minor pathogens, based on the severity of the disease they are able to cause [1]. During the past decade, culture-independent DNA-based methods have been introduced, which are now commercially available in many countries for field mastitis diagnostics [3, 5, 6]. PCR test results, which often include more species than detected in conventional culturing, have inspired discussion about the clinical relevance of the target species reported [5, 7].

A healthy mammary gland has been considered to be a sterile environment, in particular in pre-pubertal animals with intact teats before colostrogenesis and initiation of milk secretion [8]. Around the first parturition and after start of milking, the mammary gland becomes a functionally open system with a direct connection to the environment. Presence of a natural community of microbes within the mammary gland, the microbiota, has been hypothesized [9, 10]. The collective genetic composition of the microbiota is usually referred to as the microbiome [11]. The mammary gland microbiome and the microbiome of the milk can be considered to be highly similar, where the origin of microbes in the milk could be from the upper parts of the mammary gland, but it is very likely that many of these microbes migrate from extra-mammary sites and the environment [10]. In cows, milk sampling is simple compared with humans or many other animal species [12, 13]. Aseptic sampling is the recommended method to collect bovine milk samples but it can never ensure that milk samples are completely free from contaminating microbes [7, 14, 15].

The advent of high-throughput sequencing platforms has led to studies investigating microbial communities in a broad range of different biological ecosystems, including bovine milk [9]. The most common approach to explore the milk microbiome and its dynamics is sequencing of the 16S rRNA gene, which has been applied in studies on bovine mastitis [16–18]. The paradigm of IMI as an infection caused by one or maximally two microbial species has been challenged by recent studies on bovine mastitis. Along with the new microbiome data, a new hypothesis on possible “dysbiosis” of the mammary gland has been introduced as a predisposing factor for IMI and mastitis [9, 10]. Microbiota consisting of a wide selection of genera with a great microbial diversity have been found to be present in milk from quarters with mastitis [16–18]. Mastitic quarters have demonstrated a higher bacterial load than healthy quarters [19]. Most microbes reported are completely new in the phylogeny of mastitis causing microbial agents. Furthermore, the milk microbiome in bovine mammary quarters free from intramammary infection and inflammation, with a low milk somatic cell count, has been even more diverse than that seen in quarters with clinical mastitis [17, 18]. The possible clinical significance of these findings in the milk microbiome remains completely open.

In milk samples taken from quarters with pathogen-specific IMI, sequences of bacterial genera and species present in the microbiome have in general corresponded to those detected by culture [16, 17]. Some common udder pathogens such as *Escherichia coli*, *Klebsiella* spp. and *Streptococcus uberis* have been the single most prevalent microorganism in the microbiome [16]. In milk samples from IMIs due to *Trueperella pyogenes*, *Streptococcus dysgalactiae*, and *Staphylococcus aureu*s as diagnosed by culture, sequences of the same pathogens have been among the most common also in the milk microbiome [16]. Metagenomic profiling has also previously been applied to study the possible impacts of antimicrobial treatment on milk microbiota [19–22].

Culture-independent profiling of bacterial communities in the bovine mammary gland represent an approach which, while it provides a considerable amount of new information, also requires critical assessment [23, 24]. The methodologies used are very sensitive and prone to contamination or other pitfalls during sampling, sample handling and all processing steps of the analysis [25, 26]. Biological conclusions on the results of microbiome analyses must always be done with caution.

The aim of this study was to analyze the composition of bacterial communities in bovine mastitic milk samples using Next Generation Sequencing of amplicons from 16S rRNA gene and compare the microbiomes with conventional culture and PCR assay results from the same samples. Milk samples from healthy mammary quarters with no inflammatory reaction, from the same cows were also included. Samples were processed separately in two datasets and the results were compared.

## Materials and methods

### Milk samples

The sampling was carried out in Estonia during 1 day in June 2013 on a large dairy herd with 700 dairy cows. They were kept in tie stalls with concrete floors and straw bedding, and had an average milk production of 9650 kg. The herd belonged to the practice area of the Large Animal Clinic of the Estonian University of Life Sciences. The experiment was approved by the Commission of Animal Trials at the Estonian Ministry of Agriculture (No 7.2–11/1). The sampling procedure is reported in detail by Hiitiö et al. [7]. In brief, cows to be sampled were preselected based on composite milk SCC > 200 000 cells/mL in the previous DHI samples. Extremely dirty or nervous cows were excluded to avoid excessive risk of sample contamination. On the sampling day, milk CMT was performed from each quarter and cows with a CMT score ≥ 3 on a scale of 1 to 5 in at least one quarter were included in the study. In addition, healthy quarters with a CMT score 1 of the same cows were sampled.

Before sampling, the udder and the teats were cleaned with a moist towel. The teat end of the sampled quarter was wiped with cotton moistened in 70% ethanol until visibly clean and 10 mL of milk was collected in a plastic milk vial (Linkoputki 16 × 100 mm, Plastone, Mekalasi, Finland) without preservatives. The sampler wore disposable gloves. The samples were cooled immediately and transported in cooler boxes to the laboratory of the Department of Production Animal Medicine (Faculty of Veterinary Medicine, University of Helsinki) within 8 h and thereafter stored in a refrigerator at 6 °C. The following day, milk from all samples was aseptically drawn into 2.5-mL aliquots (Vacuette Tube Z, 4 mL) and stored at −20 °C for PCR analysis. Remaining aliquots were stored in similar manner for later DNA extraction for microbiome analysis.

### Conventional bacteriological culturing

The milk samples were cultured using conventional methods as described by Hogan et al. [27]. A total of 0.01 mL of milk was streaked onto blood agar and incubated at 37 °C. Agar plates were examined for growth after 18 to 24 h and after 48 h and colonies identified according to standard procedures [27].

### Milk NAGase activity determination

Milk NAGase activity was measured by a fluoro-optical method using an in-house microplate modification developed by Mattila and Sandholm [28] and further modified by Hovinen et al. [29]. NAGase activity was expressed as picomoles of 4-MU/min per microliter of milk at 25 °C. Inter-assay and intra-assay coefficients of variation for NAGase activity were 4.9 and 3.9%, respectively. The reference value for normal milk NAGase activity is 0.1–1.04 [29].

### Real-time PCR

Frozen samples were thawed and analyzed within a month from the sampling, using real-time PCR in the laboratory of Thermo Fisher Scientific Ltd. (Vantaa, Finland). PathoProof Complete-16 kit was used, which contained oligonucleotides for the staphylococcal β-lactamase gene (*blaZ*) and for microbial species or groups of species: *Corynebacterium bovis*, *Enterococcus faecalis* and *Enterococcus faecium*, *Escherichia coli*, *Klebsiella oxytoca* and *Klebsiella pneumoniae*, *Mycoplasma bovis*, *Mycoplasma* spp., *Prototheca* spp., *Serratia marcescens*, *Staphylococcus aureus*, *Staphylococcus* spp., *Streptococcus agalactiae*, *Streptococcus dysgalactiae*, *Streptococcus uberis*, *Trueperella pyogenes* and *Peptoniphilus indolicus*, and yeasts.

### DNA extraction

For practical reasons, the DNA extraction and microbiome analysis of the milk samples collected at the same day were performed in two parts. In the first part (dataset 1), 25 samples were analyzed, and in the second part (dataset 2) 24 samples. One sample was included in both datasets and could be used as an internal control of the microbiome analysis. The samples for microbiome analysis were selected based on PCR results: samples positive for major and minor udder pathogens in PCR were selected, as well as samples negative in PCR. Exclusion criteria were ≥ 3 pathogens, yeast or algae (*Prototheca*) in the sample. The best samples in this respect were included in the first dataset. In the second dataset, three samples with yeast, two with *Prototheca* algae and two ≥ 3 pathogens [*Staph. aureus*, non-aureus staphylococci (NAS) and *C. bovis*] were included to get enough samples. The DNA content of the samples varied but samples were not selected based on DNA extraction results.

The extraction of DNA from milk samples of both datasets was performed by the same experienced laboratory technician in the same laboratory in a laminar flow cabinet using PowerFood™ Microbial DNA Isolation Kit (MoBio Laboratories, Qiagen, Carlsbad, CA, USA). Set 1 DNA was extracted in November 2014 and set 2 DNA in January 2016. The amount of milk, per sample, used in DNA extraction was 1.8 mL, except for some samples, which did not contain enough milk. In these cases the remaining volume was used. The DNA concentration and purity were measured using a NanoDrop 2000 equipment (Thermo Fisher Scientific, Waltham, MA, USA). The DNA concentration, ng/μL, was measured at 260 nm and the purity was assessed using the 260 nm/280 nm and 260 nm/230 nm wavelength ratios.

### Microbiome analysis

#### Generation of 16S amplicon libraries

Extracted DNA samples were quantified using the High Sensitivity DNA Qubit system (ThermoFisher, Paisley, UK). 16S libraries encompassing the V3 and V4 regions were generated by Glasgow Polyomics. Both sample sets were processed by the same technician and used the same reagents at different times. In brief, the V3 and V4 regions of bacterial 16S were amplified using Kapa HiFi Hotstart readymix (2×) (Kapa Biosystems, Wilmington, MA, USA) with the addition of primers specific for the V3 and V4 regions of 16S (based on the standard Illumina 16S primers), which contain an overlap sequence making the primers compatible with the Nextera XT indexing reagents (Illumina, San Diego, CA, USA). Samples were then amplified using a 5 min 95 °C hotstart followed by 26 cycles of 95 °C for 30 s and 60 °C for 1 min with a final elongation step of 60 °C for 5 min.

The resulting amplicons were purified using bead extraction (SPRI select beads, Beckman Coulter, Brea, CA, USA), using 0.9× beads followed by 80% ethanol washes and resuspension in 20 μL of 10 mM Tris buffer. The amplicons were quantified using the High Sensitivity DNA Qubit system and profiles were obtained from an Agilent 2100 Bioanalyser using High Sensitivity DNA reagents (Agilent, Santa Clara, CA, USA).

Samples were then standardized to 10 ng per reaction and amplified in the presence of Nextera XT v2 indexes using Kapa Hifi Hotstart readymix (2×) for 8 cycles. The resulting indexed libraries were then purified and quality controlled as before.

#### Sequencing

The libraries were combined in equimolar ratios and sequenced on a MiSeq (Illumina, San Diego, CA, USA) instrument using a paired end, 2 × 300 bp, sequencing run. Samples were sequenced with an average of 50 000 reads per sample.

Possible contamination of reagents was controlled by running a negative control [Nuclease-Free water (Ambion™, AM9932, Thermo Fisher Scientific) instead of a DNA sample] through the whole analysis in conjunction with the samples. Water only samples were treated identically to samples. The resultant libraries were extremely low, this was significantly lower than samples by more than one order of magnitude. These libraries were not deemed suitable for sequencing due to the extremely low concentrations.

#### Analysis

FastQ files were quality filtered and trimmed using cutadapt [30] with a minimum length of 250 bp per read and a minimum quality score of 25. Paired end reads were combined using Pandaseq [31], which were then combined into a single Fasta file using the QIIME package [32]. Further processing and analysis was completed using the QIIME wrapper and its packaged software: taxonomic classification was carried out using UCLUST [33] and alignment against the Greengenes database (gg13) [34] using the PyNAST algorithm [35]. Taxonomy was assigned [36–38], and alpha rarefaction and beta diversity analyzed [39]. Taxonomy bar charts were generated [40, 41]. Samples were analyzed as individual samples and as groups. Bar charts and cladograms representing the biomarkers discovered using LDA analysis were generated with LefSe [42].

## Results

### Alpha diversity in datasets

In total, 16S rRNA gene sequences (reads) of 751 bacterial genera were detected with at least one read in one sample in either or both of the datasets, 700 in dataset 1 and 660 in dataset 2. When rare sequences with < 5 reads in the total of 25 (dataset 1) or 24 (dataset 2) samples were excluded, the number of genera in datasets 1 and 2 were 589 and 542, respectively. The number of genera with an average > 200 reads per sample was 45 and 25 in dataset 1 and 2, and the number of genera with median read number > 200 reads per sample 19 and 9 in datasets 1 and 2, respectively. Thus, most genera appeared with low read numbers and/or in few samples. The average total read number per sample was 98 711 in dataset 1 and 63 657 in dataset 2. The median of total read number per sample was 76 729 in dataset 1 and 68 364 in dataset 2.

**Figure 1:**
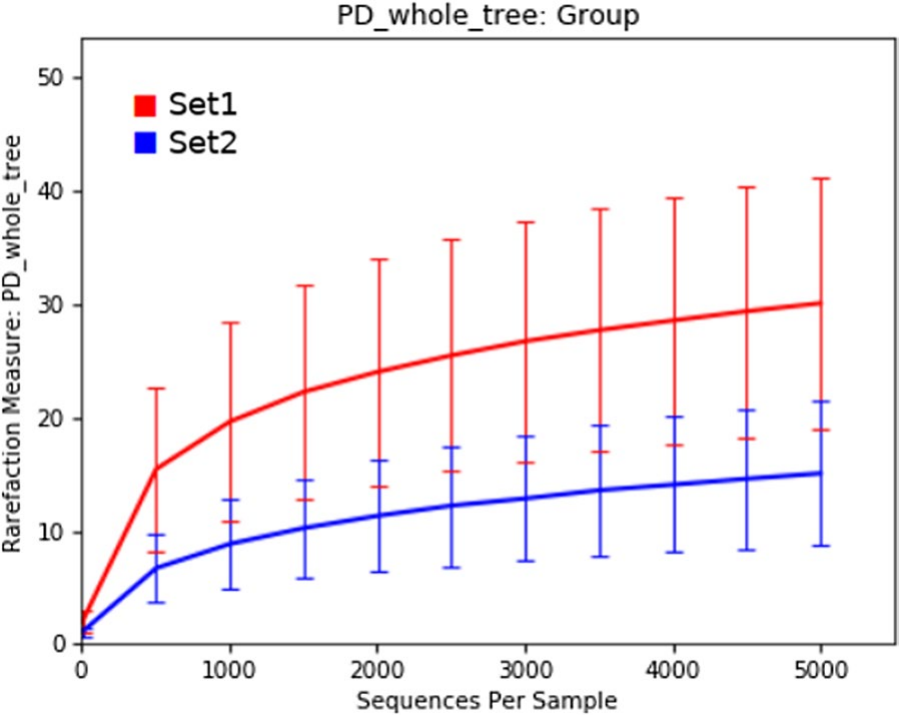
**Microbial community richness.** Alpha diversity rarefaction curves (PD whole tree) demonstrating the sizeable difference in microbial community richness between set 1 (red) and set 2 (blue). Error bars represent the intra-set variation observed.

Analysis of the alpha diversity using whole tree phylogenetic diversity (PD_whole_tree) revealed distinct differences in the species richness between the two datasets, with dataset 1 demonstrating much higher species richness than dataset 2 even at very low sub-sampling levels (Figure 1).

### The most common genera

**Figure 2:**
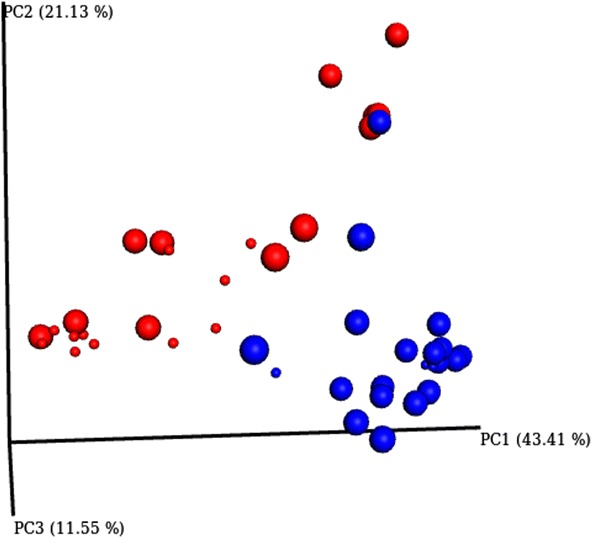
**3D Emperor PcoA plot demonstrating set variance and infection status.** Microbial community profile using beta diversity represented in a 3D Emperor plot using weighted Unifrac distances for PcoA analysis. Red dots represent set 1, blue dots represent set 2. Large spheres represent samples that were not positive for any major/minor pathogens by PCR, smaller spheres represent samples that were positive for at least one major/minor pathogen by PCR.

In dataset 1, the genera detected with highest average read numbers per sample were *Staphylococcus* (average 30 534 reads/sample, 30.9% of all reads), *Streptococcus* (average 10 432 reads, 10.6% of all reads), *Corynebacterium* (average 9303 reads, 9.4% of all reads) and *Lactococcus* (average 4402 reads, 4.5% of all reads). The high average read number of *Lactococcus* was caused by one single sample with high *Lactococcus* read number. In dataset 2, the corresponding genera were *Methylobacterium* (average 63 657 reads/sample, 50.2% of all reads)*, Staphylococcus* (average 17 574 reads, 13.9% of all reads)*, Corynebacterium* (average 15 482 reads, 12.2% of all reads) and *Calothrix* (average 10 430 reads, 8.2% of all reads). The high read number for *Calothrix* was due to one sample PCR positive for *Prototheca*, where it covered 94% of the total reads. When looking at medians per sample, the most common genera in dataset 1 were *Blautia* (median read number 2437), *Sphingobacterium* (median 1977), *Treponema* (median 1780) and *Clostridium* (median 1231), and in dataset 2 *Methylobacterium* (median 36 776), *Corynebacterium* (median 2307), *Blautia* (median 649) and *Staphylococcus* (median 487). The distinct differences between the average and median suggest that *Staphylococcus* and *Streptococcus* present a high average per sample due to a small number of samples with overwhelming abundance of these genera, indicative of an active infection. Despite significant differences between infected and non-infected quarters (*p* = 0.009) the largest differences observed were seen in the separation of dataset 1 and dataset 2. This is demonstrated by PcoA analysis which clearly defines two groups (dataset 1 and dataset 2) whilst the separation of infected vs non-infected is not very distinct through this analysis (Figure 2).

### Relation between Ct values and read numbers

When the Ct value of the PCR analysis was low indicating a high amount of pathogen DNA, the read number of the corresponding pathogen was high and the reads of that bacterial genus/species covered the majority of the total and relative amount of reads. Respectively, when the Ct value was high indicating low amount of pathogen DNA, the read number of the corresponding pathogen was smaller and the reads of that bacterial genus/species covered a smaller proportion of the total amount of reads. This relationship was seen in association with both major and minor pathogens and demonstrates the consensus between the two methodologies.

### Samples PCR positive for major udder pathogens

All samples positive for major pathogens in the PCR assay were also positive according to the conventional culture, also all negative samples were negative in both assays. Six samples PCR positive for minor pathogens with rather high Ct values did not harbor bacterial growth in the culture. PCR assay is used as the reference microbiological test in this study.

#### *Staphylococcus aureus*

In dataset 1 three samples were PCR positive for *Staph. aureus.* In PCR the cycle threshold (Ct) values for *Staph. aureus* in these samples were relatively low, < 30.0 (26.2–28.9) indicating a moderate level of *Staph. aureus* DNA. Two of the samples were also PCR positive for NAS and one for *C. bovis*. The samples had a high amount, 90 550, 175 062, and 213 389, of *Staphylococcus* reads, which covered 96%, 97% and 93% of the total amounts of reads in these samples. Of the 11 PCR negative samples, in 10 samples *Staphylococcus* reads covered 0.16% to 1.62% of the total amount of reads in these samples, and in one sample 9.68%. In this and one other sample the PCR Ct values of *Staphylococcus* spp. were > 37.0 but < 40.0, indicating a low amount of *Staphylococcus* DNA. At species level, reads of *Staph. aureus* covered 88%, 89% and 92% of all *Staphylococcus* reads. Other *Staphylococcus* species with > 100 reads up to 298 reads were *Staph. agnetis*, *Staph. chromogenes*, *Staph. devriesei*, *Staph. fleurettii*, *Staph. gallinarum*, *Staph. intermedius*, *Staph. pseudolugdunens* and *Staph. xylosus*. In the PCR negative samples, *Staph. aureus* reads covered 0 to 0.83% of the total amount of reads. The sample PCR positive for *C. bovis* in addition to *Staph. aureus* had 2816 *Corynebacterium* reads (1% of all reads). The *C. bovis* reads covered only 19% (426 reads) of all *Corynebacterium* reads. Other *Corynebacterium* species with > 100 up to 582 reads were *C. efficiens*, *C. halotolerans, C. hansenii, C. marinum,* and *C. xerosis*. The read numbers of all other genera in these three samples were low.

In dataset 2 only one sample was PCR positive for *Staph. aureus* (Ct value 26.0). This sample was PCR positive also for NAS and *C. bovis* (Ct 28.4). The level of *Staphylococcus* reads was high, 370 539, and covered 96% of the total amount of reads in this sample. In the PCR negative samples *Staphylococcus* reads covered 0.11 to 1.28% of the total amount of reads. At species level, reads of *Staph. aureus* covered 99% of the total amount of *Staphylococcus* reads. Other *Staphylococcus* species with > 100 reads up to 455 reads were *Staph. agnetis*, *Staph. chromogenes*, *Staph. devriesei*, *Staph. epidermidis*, *Staph. fleurettii*, *Staph. haemolyticus*, *Staph. intermedius*, and *Staph. pseudolugdunens.* The amount of *Corynebacterium* reads was 8154, 2% of the total amount of reads. At species level, 61% of *Corynebacterium* reads belonged to *C. bovis*. Another highly abundant species was *C. halotolerans*, 18%.

#### *Streptococcus uberis*

Only one sample in dataset 1 was PCR positive for *Strep. uberis* with a Ct value 22.8 indicating a large amount of *Strep. uberis* DNA. The total *Streptococcus* read number of this sample was 179 331 reads, which covered 96% of the total amount of reads. The next common genera *Blautia*, *Sphingobacterium*, *Treponema, Staphylococcus* and *Bacteroides* were represented with ≤ 554 reads. At species level, 95% of *Streptococcus* reads belonged to *Strep. uberis*. The next common *Streptococcus* species were *Strep. orisratti* (4%) and *Strep. bovis* (1%).

In dataset 2, similarly only one sample was PCR positive for *Strep. uberis*, but with a Ct value 34.9 indicating a low amount of *Strep. uberis* DNA. The number of *Streptococcus* reads was 1037, 11% of the total amount of reads in this sample. At species level, 97% of *Streptococcus* reads belonged to *Strep. uberis.* In this sample the genus *Methylobacterium* had the highest read number which covered 60% of the total amount of reads. All other genera were represented with low read numbers only.

### Samples PCR positive for minor udder pathogens

#### Non-aureus staphylococci

In dataset 1, four samples were PCR positive for NAS only. The amount of *Staphylococcus* reads in these samples were 190 208, 19 331, 9225 and 1419, which covered 95%, 35%, 5% and 2%, respectively, of the total amount of reads in these samples. In the PCR negative samples, only a low percentage of the total amount of reads was *Staphylococcus* (see above). The PCR positive sample for NAS with the highest *Staphylococcus* read number also demonstrated higher *Staphylococcus* DNA levels by PCR compared to the other three samples (Ct values 23.1 vs. 27.8, 28.7 and 34.6). In the sample with the lowest Ct value and highest amount of *Staphylococcus* reads, *Staph. xylosus* covered 90% of the *Staphylococcus* reads. The other samples lacked a dominating *Staphylococcus* species and the species with highest read numbers were *S. arlettae*, *S. chromogenes*, *S. cohnii*, *S. fleurettii*, *S. gallinarum*, *S. massiliensis*, *S. nepalensis*, *S. pseudolugdunensis*, *S. sciuri* and *S. xylosus* (in alphabetic order). The next common genera in these samples were *Treponema*, *Streptococcus* and *Sphingobacterium*.

In dataset 2, six samples were PCR positive for NAS only. The Ct values were all > 30.0: 31.2 to 36.6, indicating a low amount of *Staphylococcus* DNA. The number of *Staphylococcus* reads varied from 61 to 4160 and the proportion of *Staphylococcus* reads of all reads from 0.2 to 6.3%. The *Staphylococcus* species with > 100 up to 902 reads in one or more samples were *Staph. aureus*, *Staph. equorum*, *Staph. haemolyticus*, *Staph. massiliensis*, *Staph. sciuri* and *Staph. xylosus*. The most common genera in these samples were *Methylobacterium*, *Helcococcus* and *Corynebacterium*.

#### *Corynebacterium*

In dataset 1, one sample was PCR positive for *C. bovis* alone with a Ct value 29.9. The number of *Corynebacterium* reads in this sample was 86 420, which covered 53% of the total amount of reads, 164 205. *C. bovis* covered 44% of *Corynebacterium* reads, *C. halotolerans* 42%, and the rest was covered by diverse *Corynebacterium* species. This sample had 38 249 *Streptococcus* reads, 23% of the total amount of reads. Two samples were PCR positive for *C. bovis* and NAS. The Ct values for *Corynebacterium* were 28.4 and 29.7 and the read numbers of *Corynebacterium* 121 371 and 4888, respectively. The proportions of *Corynebacterium* reads in these two samples were 49% and 11%, and proportions of *Staphylococcus* reads 15% and 2%, respectively.

In dataset 2, three samples were PCR positive for *C. bovis* alone with Ct values 27.8, 29.1 and 30.3, and read number 83 936, 9160 and 98 948, respectively. In these samples *Corynebacterium* reads covered 31%, 19% and 34% of the total amounts of reads, respectively. Additionally, two samples were PCR positive for *C. bovis* and NAS, one sample for *C. bovis* and yeast, and one sample for *C. bovis*, NAS and yeast. The Ct values for all pathogens in these samples were ≥ 30.0. The average number of *Corynebacterium* reads in all 7 *C. bovis* PCR positive samples was 39 905, the proportion of *Corynebacterium* reads varied from 7 to 49%. *C. bovis* covered 50% to 73% of all *Corynebacterium* reads, and the next most common species was *C. halotolerans* 14% to 30%. The proportion of *Methylobacterium* reads in these samples varied from 42 to 67%. The proportion of *Staphylococcus* reads in the NAS positive samples varied from 1 to 11%.

### Samples from healthy quarters

In dataset 1, four samples had a negative PCR result and a milk NAGase enzyme activity < 1, indicating a healthy quarter without inflammation. The genera with highest average and median read numbers (average/median) with the proportion of the total amount of reads (%) are listed in alphabetic order: *Alicyclobacillus* 2389/2328 (2.55%), *Bacteroides* 2212/2684 (2.36%), *Blautia* 5064/4461 (5.40%), *Bradyrhizobium* 3095/2821 (3.30%), *Clostridium* 2690/2640 (2.87%), *Corynebacterium* 2525/1082 (2.69%), *Oscillospira* 2018/1164 (2.15%), *Sphingobacterium* 2856/3233 (3.05%), *Streptococcus* 3970/3836 (4.24%), and *Treponema* 2006/1702 (2.14%). Some genera had high average read numbers caused by one single sample but lower medians and are not listed here.

In dataset 2, two samples had a negative PCR result and a milk NAGase enzyme activity < 1. The genus *Methylobacterium* covered 93% of reads of these samples. The next most common genera in both samples were *Blautia* with an average of 1035 reads (0.39% of all reads), *Ruminococcus* (627, 0.24%), *Corynebacterium* (1406, 0.54%) and *Clostridium* (497, 0.19%).

In dataset 1 six samples and in dataset 2 three samples were PCR negative but had milk NAGase values > 1 and were consequently not classified as samples from healthy quarters. One sample in dataset 1 was PCR positive for *Mycoplasma* spp. with a Ct value 36.5. The microbiome profiles of these samples do not differ significantly from that of the samples from healthy quarters.

### Differences between datasets 1 and 2

**Figure 3:**
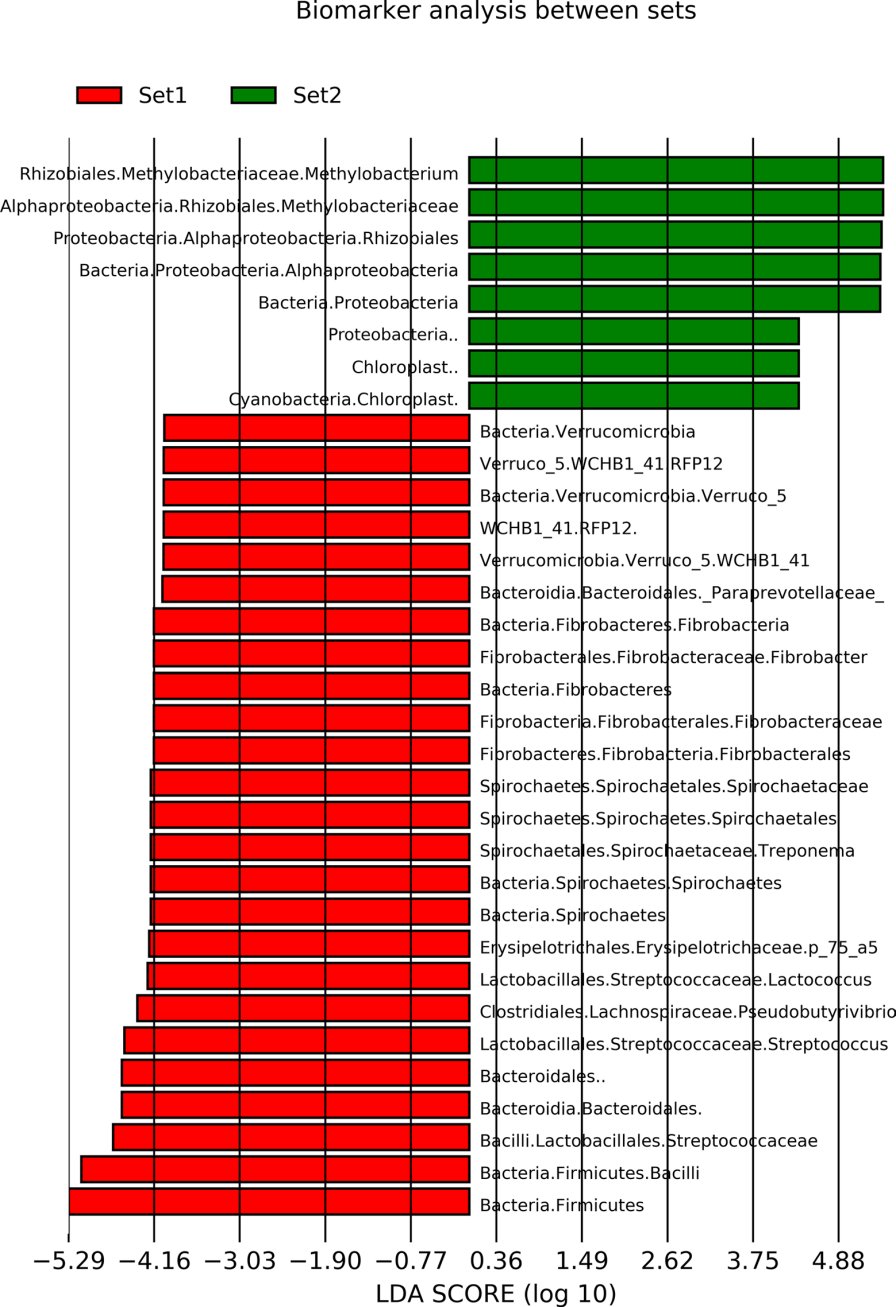
**Biomarker analysis between sets.** LefSe was used to establish the most differential taxa between set 1 and set 2. These were established with a minimum LDA score (log10) of 4 and a bonferroni corrected *p*-value < 0.001.

**Figure 4:**
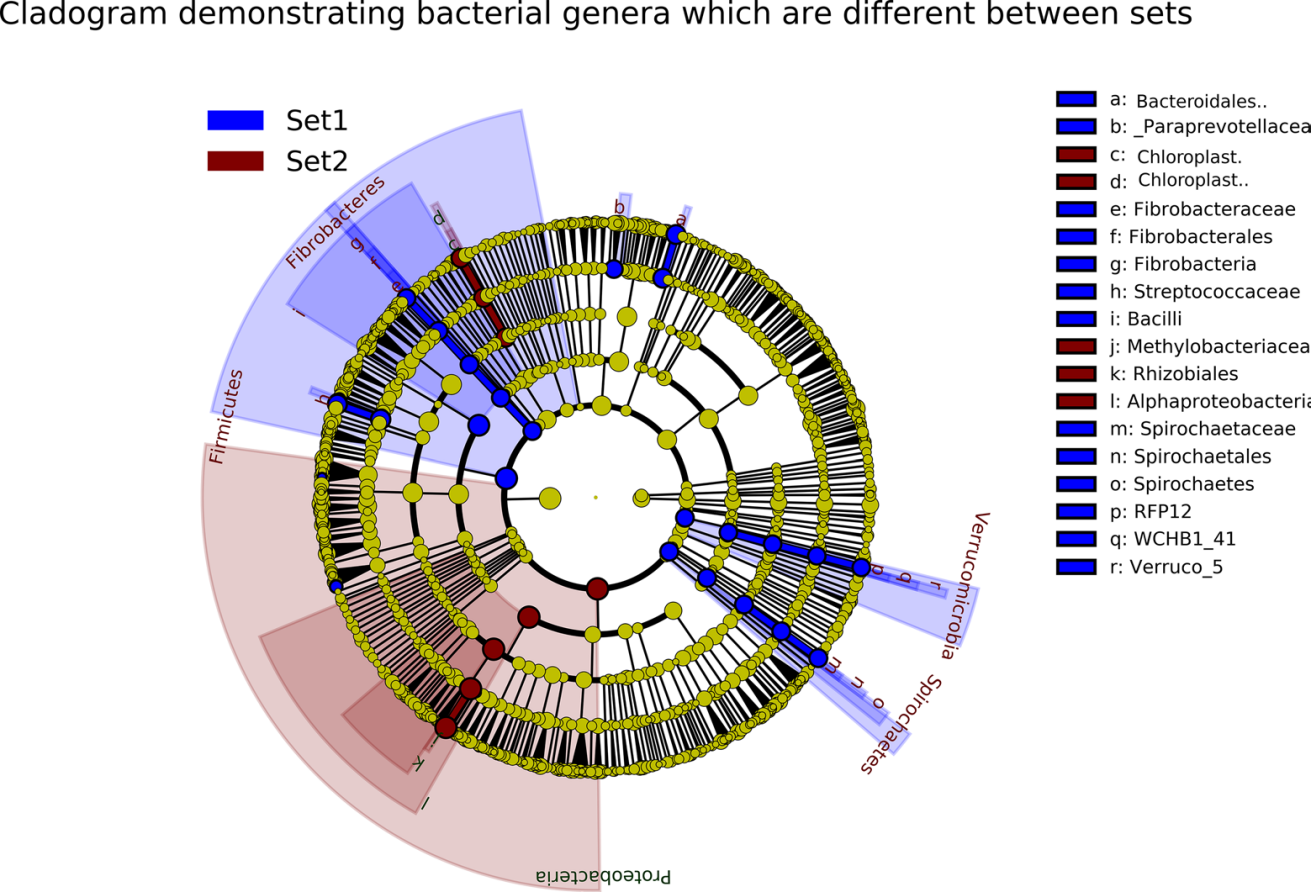
**Cladogram demonstrating bacterial genera which are different between sets.** LefSe analysis establishing the most differentially abundant taxa between set 1 and set 2 was used to generate a taxonomic cladogram demonstrating family/genera that were most discriminatory between sets. Family/genera increased in set 1 (blue) and set 2 (red) are highlighted. These had a minimum LDA score (log10) of 4, and a Bonferroni adjusted *p* value < 0.001.

The results of the microbiome analyses of datasets 1 and 2 are distinctly different, as the bacterial genera with the highest read numbers in datasets 1 and 2, especially in PCR negative samples and samples PCR positive for minor pathogens, are very different. One sample, culture and PCR positive for *Enterococcus faecalis/faecium* with a Ct value 28.6 was included in both datasets and could be used as an example of differences between datasets. Table 1 shows the bacterial genera with highest number of reads in this sample in datasets 1 and 2. Thirty genera with highest number of reads of each dataset are included in the table, and only eight of these genera belong to the top 30 in both datasets. One of these 8 genera is *Enterococcus*, for which this sample was positive in microbiological testing. Twenty genera included in top 30 in one dataset have zero reads in the other dataset. In addition, 14 of these genera have only 1 to 11 reads in the other dataset. Table 2 shows the read numbers and relative abundance of all reads of the 19 genera with highest read numbers in datasets 1 and/or 2. Linear discriminant analysis (LDA) was conducted between the two sets to identify genera/family level microbes which are significantly different between the two sets. Only microbes which were significantly different with an LDA score > 4 were included. These results reinforce the individual differences mentioned previously with *Methylobacterium* being much more common in dataset 2 compared to dataset 1. *Streptococcus* was also significantly different (Figures 3 and 4).

**Table 1:** **Microbiome results of the milk sample included in both datasets**

Genus	Reads in dataset 1	Relative abundance, %	Reads in dataset 2	Relative abundance, %
*Acinetobacter*	0	0	*337*	*0.9*
*Agrobacterium*	*487*	*1.9*	*270*	*0.7*
*Akkermansia*	*259*	*1.0*	0	0
*Alkaliphilus*	*233*	*0.9*	6	0.02
*Bacillus*	95	0.4	*824*	*2.2*
*Bacteroides*	*1177*	*4.5*	11	0.03
*Blautia*	*2477*	*9.5*	*1062*	*2.8*
*Calothrix*	*296*	*1.1*	2	0.005
*Chryseobacterium*	1	0.004	*11 426*	*30.4*
*Clostridium*	*623*	*2.4*	*318*	*0.8*
*Corynebacterium*	37	0.1	*4516*	*12.0*
*Delftia*	0	0	*499*	*1.3*
*Desulfotomaculum*	3	0.01	*1405*	*3.7*
*Desulfovibrio*	*541*	*2.1*	5	0.01
*Dysgonomonas*	*364*	*1.4*	*171*	*0.5*
*Enhydrobacter*	0	0	*884*	*2.4*
*Enterococcus*	*447*	*1.7*	*7176*	*19.1*
*Fibrobacter*	*565*	*2.2*	0	0
*Flavobacterium*	46	0.2	*443*	*1.2*
*Lachnospira*	*289*	*1.1*	104	0.3
*Lactobacillus*	*303*	*1.2*	1	0.002
*Legionella*	6	0.02	*199*	*0.5*
*Leptotrichia*	0	0	*808*	*2.2*
Candidatus *Methylacidiphilum*	*1120*	*4.3*	0	0
*Methylobacterium*	0	0	*352*	*0.9*
*Microbacterium*	0	0	*181*	*0.5*
*Mycobacterium*	0	0	*308*	*0.8*
*Neisseria*	0	0	*934*	*2.5*
*Oscillospira*	*719*	*2.8*	0	0
*Oxalobacter*	1	0.004	*262*	*0.7*
*Paludibacter*	*312*	*1.2*	103	0.3
*Parabacteroides*	0	0	*161*	*0.4*
*Parapedobacter*	*249*	*1.0*	0	0
*Paraprevotella*	*671*	*2.6*	2	0.005
*Pedobacter*	*629*	*2.4*	120	0.3
*Pelomonas*	0	0	*156*	*0.4*
*Phascolarctobacterium*	*357*	*1.4*	0	0
*Porphyromonas*	*454*	*1.7*	3	0.008
*Prevotella*	*1081*	*4.2*	*146*	*0.4*
*Propionicimonas*	0	0	*387*	*1.0*
*Pseudobutyrivibrio*	*282*	*1.1*	0	0
*Pseudomonas*	204	0.8	*349*	*0.9*
*Ralstonia*	87	0.3	*181*	*0.5*
*Ruminococcus*	*623*	*2.4*	60	0.2
*Slackia*	*312*	*1.2*	1	0.003
*Sphingobacterium*	*2587*	*10.0*	133	0.4
*Sphingomonas*	*245*	*0.9*	*142*	*0.4*
*Staphylococcus*	*466*	*1.8*	*246*	*0.7*
*Streptococcus*	*247*	*1.0*	3	0.008
*Tepidimonas*	0	0	*373*	*1.0*
*Treponema*	*2760*	*10.6*	0	0
*Trichococcus*	3	0.01	*375*	*1.0*
Total amount of reads in the sample	25,961		37,527	

The sample was positive for *Enterococcus* spp. in PCR and bacterial culture. Thirty bacterial genera with highest number of 16S reads in this sample in dataset 1 and in dataset 2, 52 bacterial genera in total, were included in this table. Only 8 genera belonged to the 30 genera with highest read numbers in both datasets. The number and relative amount of reads are italicized when belonging to the top 30.

**Table 2:** **Median read numbers and relative abundance (%) of the total read numbers of the most common bacterial genera for different sample groups in datasets 1 and 2: samples PCR positive for** ***Staph. aureus*** **and NAS/*C. bovis***, ***Strep. uberis*, minor pathogens (= NAS and** ***C. bovis*), NAS only,** ***C. bovis*** **only, and samples from healthy quarters (PCR negative and NAGase value < 1)**

Genus	Dataset 1, groups by PCR result						Dataset 2, groups by PCR result					
	3 *Staph. aureus* Read no/%	1 *Strep. uberis* Read no/%	7 Minor Read no/%	4 NAS Read no/%	1 *C. bovis* Read no/%	4 Neg. NAG < 1 Read no/%	1 *Staph. aureus* Read no/%	1 *Strep. uberis* Read no/%	14 Minor Read no/%	6 NAS Read no/%	3 *C. bovis* Read no/%	2 Neg. NAG < 1 Read no/%
*Alicyclobacillus*	97/0.05	3/0.00	173/0.11	68/0.05	1434/0.87	2328/2.48	0/0	0/0	0/0	0/0	0/0	0/0
*Alkaliphilus*	28/0.02	74/0.04	1155/0.70	391/0.30	2334/1.42	1691/1.80	163/0.04	43/0.44	201/0.23	251/0.37	334/0.12	160/0.06
*Bacteroides*	122/0.07	318/0.17	1526/0.93	2053/1.55	757/0.46	2683/2.86	9/0.00	77/0.79	140/0.16	31/0.05	696/0.26	216/0.08
*Blautia*	331/0.18	554/0.30	2809/1.71	3495/2.64	1023/0.62	4461/4.76	19/0.00	386/3.96	634/0.71	707/1.03	870/0.32	1034/0.39
*Bradyrhizobium*	212/0.12	19/0.01	706/0.43	71/0.05	1996/1.22	2821/3.01	0/0	10/0.10	6/0.01	9/0.01	7/0.00	13/0.00
*Clostridium*	208/0.12	295/0.16	1469/0.89	1594/1.20	1371/0.83	2039/2.18	259/0.07	6/0.06	318/0.36	309/0.45	801/0.30	496/0.19
*Corynebacterium*	25/0.01	142/0.08	3383/2.06	173/0.13	86 420/52.63	1081/1.15	8154/2.11	261/2.68	2705/3.04	2493/3.65	83 936/31.10	1406/0.54
*Fibrobacter*	62/0.03	301/0.16	941/0.57	1418/1.07	422/0.26	1352/1.44	0/0	2/0.02	0/0	0/0	17/0.01	32/0.01
C*. Methylacidiphilum*	158/0.09	221/0.12	818/0.50	1737/1.31	430/0.26	1204/1.28	0/0	0/0	0/0	0/0	42/0.02	124/0.05
*Methylobacterium*	18/0.01	13/0.01	104/0.06	35/0.03	686/0.42	502/0.54	2472/0.64	5840/59.92	43 264/48.57	46 816/68.48	124 470/46.12	245 445/93.49
*Oscillospira*	82/0.05	157/0.08	644/0.39	678/0.51	497/0.30	1163/1.24	10/0.00	0/0	122/0.14	91/0.13	214/0.08	386/0.15
*Paraprevotella*	83/0.05	147/0.08	521/0.32	1061/0.80	212/0.13	857/0.91	0/0	0/0	1/0.00	0/0	231/0.09	54/0.02
*Pedobacter*	73/0.04	101/0.05	633/0.39	869/0.66	290/0.18	1045/1.12	2/0.00	3/0.03	34/0.04	9/0.01	300/0.11	148/0.06
*Prevotella*	102/0.06	245/0.13	415/0.25	1368/1.03	366/0.22	1156/1.23	1/0.00	0/0	25/0.03	13/0.02	0/0	71/0.03
*Ruminococcus*	59/0.03	117/0.06	907/0.55	939/0.71	488/0.30	1678/1.79	43/0.01	58/0.60	295/0.33	165/0.24	1299/0.48	626/0.24
*Sphingobacterium*	195/0.12	519/0.28	2529/1.54	3123/2.36	932/0.57	3233/3.45	0/0	1/0.01	30/0.03	2/0.00	169/0.06	54/0.02
*Staphylococcus*	175 062/97.3	378/0.20	9225/5.62	14 278/10.79	1311/0.80	869/0.93	370 539/95.88	12/0.12	664/0.75	785/1.15	233/0.09	397/0.15
*Streptococcus*	353/0.20	179 331/96.1	2143/1.31	1510/1.14	38 249/23.29	3835/4.09	645/0.17	1037/10.64	3/0.00	1/0.00	91/0.03	68/0.03
*Treponema*	234/0.13	415/0.22	2017/1.23	2825/2.13	674/0.41	1701/1.82	11/0.00	0/0	0/0	1/0.00	72/0.03	1/0.00
Reads total	179 938	186 696	164 205	132 326	164 205	93 700	386 450	9746	89 072	68 364	269 890	262 526

The number of samples in each category is indicated.

## Discussion

The present study makes one more contribution to the complex issue on the microbiome of bovine milk, with some critical remarks. In our raw data, the total number of genera detected in the milk samples was over 700, if all findings starting from one read, were reported. However, findings with very low reads are unreliable and some cut-off for minimum reads should be used. In general singlets should be discounted to ensure that results are not sporadic. In fact, the data presented here suggests that there is a strong basis for discounting low read numbers due to differences between batches or sets. Studies on milk microbiome published so far have not given threshold for read numbers to be significant, which makes comparisons difficult. An important finding here was that results of the two datasets originating from the same sampling performed during 1 day on one single herd differed, as some genera were present in all samples in one dataset but not detected in the other. The genera detected solely in one dataset are very likely post-sampling contaminants which have appeared at some stage of the sample processing and analysis.

The most common phyla detected in mastitic milk samples in the present study, *Firmicutes*, *Actinobacteria*, *Bacteroides*, and *Proteobacteria*, were the same as have been reported in previous studies [21, 43]. Results from earlier studies have been contradictory. In the first published study on the mastitic milk microbiome, a high number of anaerobic bacterial sequences from genera *Fusobacterium* and *Porphyromonas*, with sequences belonging to *Fusobacterium necrophorum* were highly prevalent in all mastitic samples [16]. In this study, *F. necrophorum* sequences were practically absent in healthy, low SCC quarters, in contrast to our study, where genus *Fusobacterium* was present also in healthy quarters. In two studies, samples from quarters with clinical mastitis, with no growth in culture, were compared with samples from healthy quarters [17, 18]. Kuehn et al. [17] found significantly more *Brevundimonas*, *Burkholderia*, *Sphingomonas*, and *Stenotrophomonas* in mastitic samples. In another study the genus *Sphingobacterium* was significantly more abundant in quarters with increased milk SCC, as was *Streptococcus* [18].

It has been speculated that certain genera would represent the “natural” microbiome of bovine milk [9, 10, 17, 18]. Logically, these genera should be present abundantly in milk from healthy quarters. Indeed, it has been reported that microbiota profiles from healthy quarters could clearly be discriminated from mastitic samples [18]. In our study the microbiota profiles of milk of mastitic and healthy quarters differ as well, but the bacterial genera detected in samples of healthy quarters differ also between the datasets 1 and 2. In the study by Oikonomou et al. [18] four bacterial genera were present in all samples from healthy quarters: *Faecalibacterium* spp., unclassified *Lachnospiraceae*, *Propionibacterium* spp. and *Aeribacillus* spp.; also *Nocardiodes* and *Paenibacillus* were more abundant in healthy quarters [18]. *Propionibacterium* spp. were present in all healthy quarters, and at species level, *P. acnes* was the most prevalent bacterium in the majority of milk samples from healthy quarters with low SCC, which led the authors to speculate the possible role of these bacteria as “natural microbiota” of healthy quarters [18]. The abundance and thus relative proportion of *Propionibacterium* was very low in our samples. In the same study [18], *Strep. uberis* was detected in all groups of samples, and was proposed to possibly belong to the natural microbiota of the milk [18]. Our study did not support this as *Streptococcus* sequences were abundant in one dataset but almost lacking in the other, indicating possible contamination in one dataset. In the study by Ganda et al. [19], the dominating genera in milk samples taken from healthy quarters were different from reported in our study or in the other studies.

In one study where effects of dry cow therapy on the microbiota of healthy quarters were investigated, the most abundant genera were *Corynebacterium*, *Acinetobacter*, *Arthrobacter*, *Staphylococcus*, and *Psychrobacter* [21], i.e. different genera than reported in the other cited studies. Antimicrobial dry cow therapy had no effects on milk microbiome. Contradictory findings were reported in another study [22], where bacterial genera of the phylum *Proteobacteria* increased in the colostrum samples after dry cow therapy using a combination of penicillin and novobiocin. In that study, the phylum *Firmicutes* including the genus *Butyrivibrio*, and unclassified families *Clostridiaceae* and *Bacillales*, were the main bacteria in milk microbiota of healthy quarters before drying-off, which differs from previous studies. Lima et al. [43] studied microbiomes in colostrum samples of dairy cattle, finding *Staphylococcus*, *Prevotella*, *Ruminococcaceae*, *Bacteroidales*, *Clostridiales*, and *Pseudomonas* as the dominating genera. At family level, the most abundant families in samples from healthy quarters of twelve cows in an experimental mastitis study were *Ruminococcaceae* (mean 16.8%), *Lachnospiraceae* (mean 7.0%), *Aerococcaceae* (mean 6.8%), *Enterobacteriaceae* (mean 6.3%), *Planococcaceae* (mean 5.7%), *Bacteroidaceae* (mean 5.4%), *Corynebacteriaceae* (mean 5.1%), *Clostridiaceae* (mean 4.2%), *Bacillaceae* (mean 3.5%), and *Staphylococcaceae* (mean 2.8%) [20]. As seen above, studies on milk microbiome published so far have variable and often contradictory results.

It has been suggested that IMI would be a consequence of a dysbiosis of the mammary gland microbiome, and not merely an invasion of pathogenic bacteria from outside the gland [9, 10]. This hypothesis has been based on studies previously referred to, where microbial communities of samples originating from healthy quarters have differed from those of mastitic samples. No direct scientific evidence for the dysbiosis theory has been published. Another, maybe more likely explanation for the differences between microbiomes in mastitic and healthy quarters could be the changed composition of milk in mastitis. Mastitic milk and whey favor growth of different bacterial genera as compared with normal milk [44–46]. Species not belonging to udder pathogens like lactobacilli, *Bacillus subtilis* and *Pseudomonas fluorescens* have been inhibited by mastitic milk, whereas growth of known pathogens such as *Staph. aureus* and *E. coli* has been weaker in normal milk [44].

Microbiota present in milk samples drawn from mastitic or healthy quarters do not represent the whole mammary gland but just milk from teat cistern and possibly milk chamber of the udder, and we would not call the milk microbiome the same as the microbiome of the mammary gland. Microbiota in milk samples consist of microbes coming from an infected gland or extramammary sites or both, and also hypothetically, microbes belonging to the so-called natural microbiota of the mammary gland. To date, no scientific evidence on the presence of a natural microbiome in a healthy mammary gland is available [23]. In IMI the mammary gland is infected and microbes can be present in the duct system and other compartments of the gland, depending on the invasiveness of the pathogen, duration of IMI, and other factors [47]. In a lactating cow, the udder is an open system, and bacteria enter the gland via the teat canal. They can be transferred during milking, in particular during inappropriate changes in the vacuum level [48]. Bonsaglia et al. [21] reported a higher bacterial load in milk microbiomes of samples taken at day 7 post-partum than in those taken at drying off, which may indicate the effect of milking on the milk microbiota. In a study investigating the impact of experimental mastitis treated with antimicrobials on milk microbiota, the authors concluded that the mammary gland would have a resilient microbiome which is established after the exposure to antimicrobials [20]. This is logical, assuming that milk microbiota consists of microbes from outside of the gland, where they reflect bacterial genera present on body sites and the environment of the cow.

It is difficult to find support for the hypothesis on some metagenomic profile which would reflect a “normal” microbiome of the milk. During the milk sampling, contamination of the sample can occur at many stages of the procedure. Aseptic technique is the prerequisite for taking good quality quarter milk samples [15]. However, in dairy farm conditions milk sampling is always challenging, because there are many sources of contamination like cubicles, faeces, forage, barn air etc. [14, 15]. Metzger et al. [49] found that the composition of bacterial community in milk samples differed between cows kept on different beddings. This indicates that the environment affects milk microbiomes even if milk is collected directly from the gland cistern as done in this study.

Among genera detected in milk samples, many belong to phyla and genera known to belong to the core microbiome of the bovine rumen, like *Prevotella*, *Butyrivibrio*, *Ruminococcus*, *Lachnospira*, and *Clostridium* [50, 51]. Their presence in the milk samples is not surprising as bacteria from the rumen also end up in faeces and on external body sites and the environment of the cow. Many genera reported in the milk like *Arthrobacter*, *Acinetobacter*, and *Psychrobacter* are environmental bacteria which can be detected in water, soil and other diverse habitats [52]. Despite thorough cleaning of the teat end prior to sampling, the teat canal and skin harbor bacteria which easily can contaminate the sample. Non-aureus staphylococci and *Corynebacteria* are the most likely species in this respect [7, 53, 54]. Our samples were taken on a large dairy farm with stanchion barns and straw bedding, where hygienic conditions were not optimal. Geographical conditions and sampling sites certainly have an impact on the microbiome detected in milk samples, and probably at least partially explain the very different microbiomes reported in various studies. This is supported by the study by Oikonomou et al. [18], who showed that discriminant analysis models could identify samples originating from different farms based on their microbial profiles. An interesting detail in our results was abundance of *Calothrix* in one mastitic sample positive for *Prototheca*. This has also been reported by other authors [55], who suggested that the finding may be related to environmental factors or host immunity.

In the present study, milk samples originating from mastitic quarters, the bacteria diagnosed as the cause of mastitis with multiplex real-time PCR and culture dominated the results of the microbiome analysis. The presence of pathogenic bacteria in the microbiome was highly consistent with PCR and culture results. This is in line with previous studies where mastitis has been diagnosed using conventional culturing [16, 19]. When a major mastitis pathogen *Staph. aureus* or *Strep. uberis* was detected by PCR in the milk sample with a low Ct value for that pathogen i.e. a high amount of DNA, the read numbers of these pathogens were high and formed the overwhelming majority of read numbers in these samples. The same was true for minor pathogens NAS or *C. bovis*. On the contrary, if the Ct value for a minor pathogen was high in the PCR test, indicating low levels of DNA, the read number of that pathogen was also low and covered only a small proportion of the total read numbers in the sample. Possible explanations for this phenomenon in quarters with low levels of minor pathogen DNA using PCR could be that the detected minor pathogens would origin from the teat canal and skin, which also harbor large amount of microbiota belonging to diverse, often anaerobic, genera, which are then also seen in the microbiota. In these samples, as well as in the samples from healthy quarters, bacterial genera such as *Methylobacterium* and *Treponema*, appeared with high read numbers. One explanation for the larger amount of diverse genera in PCR negative samples from healthy quarters could be that when the milk sample lacks DNA of udder pathogens or their level is low, other genera, originating from other sources like teat orifice, barn air or environment, and laboratory sources during sample preparation, DNA extraction etc., have “space” during the amplification process in the microbiome analysis, i.e. have no competition and can be amplified in excess. For example, in dataset 1, the average proportion of *Treponema* is 2.4%. In the three *Staph. aureus* positive samples the proportion of *Treponema* reads vary from 0.07 to 0.2%, and that of the *Strep. uberis* positive sample is 0.2%. In the sample PCR positive for NAS with Ct value 23.1 the proportion of *Treponema* reads is also low, 0.03%. The same for example with the genus *Blautia*, with an average proportion of 3.1% per sample. In the three *Staph. aureus* samples the proportion of *Blautia* reads were 0.1%, 0.1% and 0.2%, in the *Strep. uberis* sample 0.3% and in the NAS sample 0.02%. Similarly in dataset 2, the average proportion of *Methylobacterium* reads is 50% of all reads, but 0.6% in the *Staph. aureus* positive sample. It has been shown that samples with originally low biomass and no clearly dominating genera, contaminating organisms can comprise the majority of total sequences in the microbiome analysis [25, 52]. Milk samples from healthy quarters and quarters PCR positive with high Ct values could represent this kind of low biomass samples.

Our two datasets clearly differed in that some genera, for example *Alicyclobacillus*, *Bacteroides*, Candidatus *Methylacidiphilum*, *Fibrobacter*, *Methylobacterium*, *Paraprevotella*, *Sphingobacterium*, and *Treponema* were present in one but almost or totally lacking in the other dataset. In addition, the genus *Streptococcus* was present with high read numbers in most of the dataset 1 samples but in dataset 2 was only seen in the *Strep. uberis* positive sample. Most of these genera match with water and soil associated bacterial genera reported to contaminate samples in sequence-based microbiome analyses [52, 56, 57]. Contamination can occur during any step of sample handling and analysis, for instance laboratory reagents or kits have been shown to be contaminated at least in some cases [52]. In dataset 2, methylobacteria were detected in all samples and in most of them with high read numbers. The mean *Methylobacterium* spp. read number per sample was 63 656 (median 36 776) reads (min 352, max 261 148). The *Methylobacterium* read numbers were approximately 100-fold higher in samples from healthy quarters compared with samples with major mastitis pathogens. In dataset 1 methylobacteria were not common: the mean read number per sample for different *Methylobacterium* species was only 321 (median 90) reads (min 0, max 3355 reads). The milk sample analyzed in both datasets but with different results for a substantial part of bacterial genera detected (Table 1) raises questions about the consistency of microbiome analytics on milk samples. The DNA extraction of sets 1 and 2 were performed at different times and using different batch of the DNA isolation kits. It is likely that contamination during some step of the process has occurred. This is not exceptional, but a common and difficult to avoid problem in sensitive next-generation sequencing analyses [25].

We conclude that a critical assessment is necessary for assessing the results of milk microbiome analyses. What are the roles of the numerous different genera and species detected in the milk, are they endogenous or invaders, pathogens, commensals or contaminants? For a true understanding of the role and significance of the microbiota in the mammary ecosystem more research on their presence and dynamics in health and disease, as well as in different environments and production systems of dairy cattle, is necessary. Furthermore, sampling and analyses should be carried out according to the best practices agreed for 16S microbiome research.

## Data Availability

Data are available from the authors on request.
